# Analytical Techniques & Applications of Metabolomics in Systems Medicine and Systems Biotechnology

**DOI:** 10.5936/csbj.201301001

**Published:** 2013-02-18

**Authors:** Silas G. Villas-Boas

**Affiliations:** a Centre for Microbial Innovation, School of Biological Sciences, the University of Auckland, 3A Symonds Street, Auckland 1142, New Zealand

Metabolomics – the unbiased and non-targeted analysis of cell metabolites in biological samples – can be considered a paradigm shift in the analytical biochemistry field that took place in the post genomic era. The analysis of metabolites has been part of life science for several decades, playing very important roles in the development of methods for diagnostics of diseases, phenotypic characterisation of strains and cultivars, quality control of food products, and to study the physiological state of different organisms. However, with the advances in the technology applied to analytical chemistry combined with the genomic revolution where the genome sequence of whole biological systems became available, forced us to review the way metabolites were analysed in biological samples.

Metabolite analysis has been classically a targeted analytical approach where a defined list of compounds is analysed, ignoring all non-targeted ones. In classical metabolite analysis the sample preparation are usually extensive in order to enrich the samples with the targeted metabolites to be analysed, at the same time eliminating most interferences and non-targeted compounds. This makes targeted metabolite analysis a very robust, reproducible, quantitative and accurate approach. However that reduces tremendously our chances of making new discoveries because we significantly bias our results towards the choice of metabolite we target in a biological system. Furthermore, classical targeted metabolite analysis is usually limited to the analysis of a small number of compounds, which usually fails to meet the needs of system-wide studies. A true metabolomics approach, on the other hand, aims to develop unbiased and non-targeted methods for analysis of metabolites, favouring reproducibility, scope broadness and sensitive detectors with high specificity such as mass spectrometry (MS) and nuclear magnetic resonance (NMR) spectroscopy.

The analytical techniques developed for metabolomics are usually referred to as “comprehensive metabolite profiling”, which can be defined as the set of all metabolites or derivative products (identified or unknown) detected by analysing a sample using a particular analytical technique, together with an estimate of quantity [1]. Usually, this includes efficient chromatographic separations, but resolution can also be achieved using powerful detectors alone (e.g. NMR, MS) [1]. These techniques usually allow the detection, the identification and sometimes also the absolute quantification of dozens to hundreds of metabolites within a single analysis, which make them the ideal counterpart to genomics and proteomics and essential component to any system-wide research study. By being a non-targeted approach, the analytical techniques in metabolomics significantly increase the rate of discoveries and, therefore, they became a powerful hypotheses-generating tool in modern science. However, it is difficult to be non-targeted and present broad scope without losing accuracy and precision. Thus, through metabolomics we can only propose new hypotheses that will have to be eventually proved or disproved by carrying out further experiments often linked to a targeted analytical approach. Nonetheless, the number of hypotheses that can be generated through a single metabolomic study can be enormous, which in turn has a very positive impact on biodiscovery.

All system-wide approaches aim to explain the functionality of a biological system in terms of concerted interactions between numerous biochemicals [2]. Within this scenario, metabolomics provide critical tools to assess and characterise the numerous interactions between the cell's biochemicals. Metabolomics combined with genomics data permit the identification and characterisation of metabolic reactions, which can be used in the reconstruction of metabolic networks [2]. When metabolomics is combined with gene-expression (transcriptomics) and protein level (proteomics) data, it can be used to build and test quantitative and integrative models of cell metabolism [2]. This means that the behaviour of a biological system can be elucidated by analysing the behaviour of a mathematical model, which metabolomics plays a key role in integrating the different components of these models.

In summary, this special issue is intended to highlight both new techniques developed for metabolome analysis and the growing interest in applying these techniques to systems medicine and systems biotechnology. Given the dynamism of this relatively new field and its recognised potential for application to system-wide biological studies, this issue could not have been timelier.
